# Rheumatoid Arthritis Co-relation with Anti-CCP Antibodies with special reference to its Prevalence in Asymptomatic First-Degree Relatives

**DOI:** 10.31138/mjr.33.1.42

**Published:** 2022-03-31

**Authors:** Pramod GR, Prasanta Dihingia, Anshu Kumar Jha, Akash Gadgade, Deepti Agarwal

**Affiliations:** 1 Department of Nephrology, S.S. Institute of Medical Sciences, Davangere, Karnataka, India,; 2 Department of Medicine, Assam Medical College and Hospital, Dibrugarh, Assam, India,; 3 Department of Cardiology, Assam Medical College and Hospital, Dibrugarh, Assam, India,; 4 Senior Manager, Medical and Scientific Affairs, Navitas Life Sciences,; 5 Department of Medicine, S.S. Institute of Medical Sciences, Davangere, Karnataka, India

**Keywords:** rheumatoid arthritis, asymptomatic first-degree relative, anti-cyclic citrullinated peptide antibody

## Abstract

**Objectives:**

Rheumatoid Arthritis (RA) is a chronic inflammatory autoimmune disease. First-degree relatives (FDR) of patients with RA sharing genetic and environmental risk factors for RA may represent a pre-RA state. This study showed the clinical co-relation of RA with Anti-Cyclic citrullinated peptide (anti-CCP) antibody and prevalence of sero-positive anti-CCP antibody in asymptomatic first-degree relatives (AFDR) of rheumatoid arthritis patients.

**Methods:**

Total 85 RA patients, 105 AFDR, and 105 healthy controls who belonged to the same geographical area having no family history of autoimmune diseases were enrolled in this cross-sectional study. RA patients were clinically examined, and DAS-28 was calculated. Anti-CCP was sent for RA patients, AFDR, and control group. Appropriate statistical tools were applied to find if any significant co-relation exists.

**Results:**

DAS 28 co-related significantly with anti-CCP positivity (p≤0.01) but not with Rheumatoid Factor (RF). No significant co-relation was observed between anti-CCP and extra-articular manifestation (EAM) (p≥0.05). Seropositivity for anti-CCP antibody was detected in 22/105 (20.9%) AFDR and in 13/105 (12.3%) control group respectively. Anti-CCP antibody seropositivity was more prevalent in AFDR than in control group but the difference was not statistically significant (p = 0.1378).

**Conclusions:**

Anti-CCP should be preferred over RF as it correlated well with disease activity, but it does not guide well for the EAM. The higher sero-prevalence of Anti-CCP in AFDR may lead to higher risk of development of RA in near future. Thus, all AFDR should be screened so that we may follow up the positive cases for early detection and treatment of RA.

## INTRODUCTION

Rheumatoid arthritis (RA) is a chronic inflammatory autoimmune disease characterised by the proliferation of synovial lining cells, angiogenesis, and infiltration of mononuclear cells, resulting in joint destruction and functional disability.^1^ Although the pathogenesis of RA has not been clearly determined, it is now accepted that the development of RA is closely associated with diverse genetic factors such as the human leukocyte antigen (HLA)-DR alleles encoding the “shared epitope” and polymorphisms in potent genes, including those for protein tyrosine phosphatase, non-receptor type 22 (PTPN22), signal transducer and activator of transcription 4 (STAT4), and 6q23, as well as environmental factors such as coffee consumption and smoking.^2,3^ In addition, various other familial and twin studies have shown that the occurrence of RA has strong familial aggregation.^4^ Kolfenbach et al. suggested a model for the development of RA in three serial phases: 1) an initial genetic risk phase, 2) pre-clinical autoimmunity, and 3) clinical disease.^4^ Interactions between environmental factors and genetic risks lead to asymptomatic and pre-clinical autoimmunity characterised by early detection of RA-related autoantibodies, in susceptible individuals, like rheumatoid factor (RF) and anti-cyclic citrullinated peptide antibody (Anti-CCP antibody) and markedly increased levels of pro-inflammatory markers. Investigations showed that the presence of auto antibody positivity may precede clinically apparent RA by two decades.^5^ Moreover, studies have shown that anti-CCP is positive even in those RA patients who are RF negative. This shows that anti-CCP should ideally be used as first line investigation in RA patients.^6^ More prevalent expression of autoantibodies such as RF, RA-associated nuclear antigen, and anti-CCP antibody has been reported to occur in unaffected relatives of RA families.^7^ Therefore, first-degree relative (FDR) of patients with RA represents a high risk of cohort for future development of RA. As no study in India has ever seen the positivity rate of anti-CCP in asymptomatic first-degree relatives (AFDR), this study was undertaken.

In this study, we co-related the symptoms of RA with respect to Anti-CCP antibodies and studied the prevalence of anti-CCP antibody in AFDR of RA patients.

## MATERIAL AND METHODS

This study was a hospital-based cross-sectional study conducted on all the cases of Rheumatoid arthritis who attended Rheumatology clinic, and their accompanying AFDR over period of one year from June 2013 to June 2014. Clearance was given by the institutional ethical committee to carry out the study. The spectrum of FDRs in our study included parents and offspring of RA patients. All patients over18 years of age and classified by the 2010 Rheumatoid Arthritis Classification Criteria of American College of Rheumatology/European League Against Rheumatism Collaborative Initiative attending the rheumatology OPD or admitted in the hospital were included in the study. For AFDR, we excluded the FDRs if they had any symptoms or any history, physical examination, or laboratory findings suggestive of connective tissue disorder. Healthy controls were the apparently healthy individuals not having any collagen vascular disorders or family history of collagen vascular disorders or any disease thereof. Sampling was done by systematic sampling method. Finally, 85 patients of RA, 105 AFDR, and 105 healthy controls were recruited for the study. Thorough general and systemic examination was done in all RA patients and presence of extra articular manifestations were noted. Disease activity was calculated using DAS28 score. Apart from routine blood investigations, RF and Anti-CCP were sent for all patients and their AFDR. The ARCHITECT Anti-CCP assay was used, which is a chemilumines centmicroparticle immuno assay (CMIA) for the semi-quantitative determination of the IgG class of autoantibodies specific to cyclic citrullinated peptide (CCP) in human serum or plasma. Anti-CCP antibodies were detected by ELISA, using a commercial kit (Omega Genesis EDRA bioMerieux, Cambridgeshire, UK) and according to the manufacturer’s instructions, expressed in optical density (OD) along y-axis and corresponding antibody titre of the standards, plotted along x-axis of the curve. RF assay was done by Latex Agglutination Slide Test with RHELAX-RF reagent Kit (Tulip Diagnostics (P) Ltd. India) and subsequently the sera were examined for IgM Rheumatoid Factor by Autostat^TM^II Rheumatoid Factor IgM ELISA Kit (Hycor Biomedical, Garden Grove, California, USA). The tests were done according to manufacturer’s instructions. Level of 6.25 U/ml was taken as the standard cut-off for diagnosing a patient to be either Anti-CCP positive or negative.

Statistical analysis was done using appropriate software. Results on continuous measurements were presented in Mean ± S.D and results on categorical measurements were presented in number. The significance of study parameters between two or more groups was assessed using Fisher’s Exact test for parameters on categorical scale, and unpaired t test for parameters on continuous scale. Significance was assessed at 5% level of significance (p<0.05).

## RESULTS

Out of 85 RA cases, 33% cases belonged to the 40–50 years age group (mean age 42.81 ± 13.01 years) and male:female ratio was 1:3.7. Twenty-five patients (29.41%) had a disease duration of above 2–5 years. Duration of RA in the present study varied between 2 months to 15 yrs. Mean duration of disease was 4.05 ± 3.55 years. As expected, 89% subjects had symmetrical joint involvement and 81% had morning stiffness. Most of our patients (53%) had moderate disease activity according to DAS28 criteria and 43% had severe disease activity. Mean disease activity was 4.97±1.03. Anti-CCP positivity was seen in 71 out of 85 patients (84%) and mean ACR/EULAR joint score in them was 2.58 as compared to 24 patients with negative anti-CCP in whom joint score was 0.67 (p < 0.0001).

Disease activity with respect to anti-CCP antibody and RF has been shown in **Table 1**.

**Table 1.** Correlation of disease activity with respect to Anti-CCP antibodies, rheumatoid factor, erosive disease and extra-articular manifestation.

**DAS 28**	**Anti-CCP**				**Rheumatoid Factor**				**Erosive disease**				**Extra-articular manifestation**				**Total (N_1_ + N_2_ or N_3_ + N_4_ or N_5_ + N_6_or N_7_ + N_8_)**
	**Positive**		**Negative**		**Positive**		**Negative**		**Present**		**Absent**		**Yes**		**No**		
	**N**_1_	%	N_2_	%	N_3_	%	N_4_	%	N_5_	%	N_6_	%	N_7_	%	N_8_	%	
<2.6	01	100	0	0	0	0	01	100	0	0	1	100	0	0	1	100	1
>2.6 - <3.2	02	100	0	0	0	0	02	100	0	0	2	100	2	100	0	0	2
>3.2 - ≤5.1	32	71	13	29	31	69	14	31	20	44	25	56	16	36	29	64	45
>5.1	36	97	01	3	30	81	07	19	28	76	09	24	18	49	19	51	37
**Total**	71		14		61		24		48		37		36		49		85

* N stands for the number of patients in that particular group.

**Figure 1.**
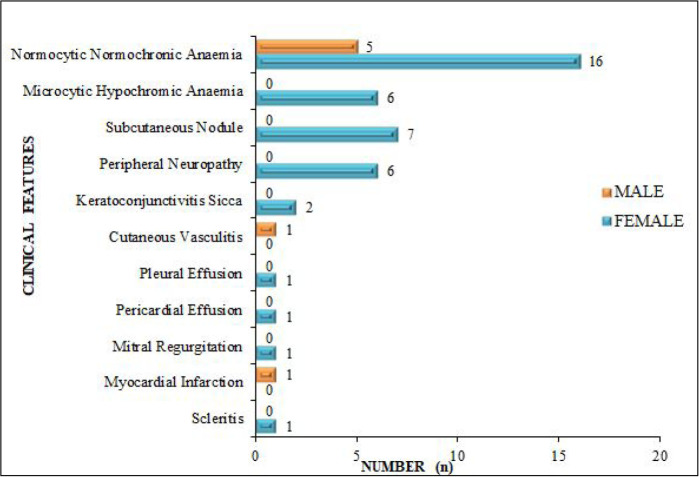
Extra-articular manifestations along with their sex distribution.

**Figure 2.**
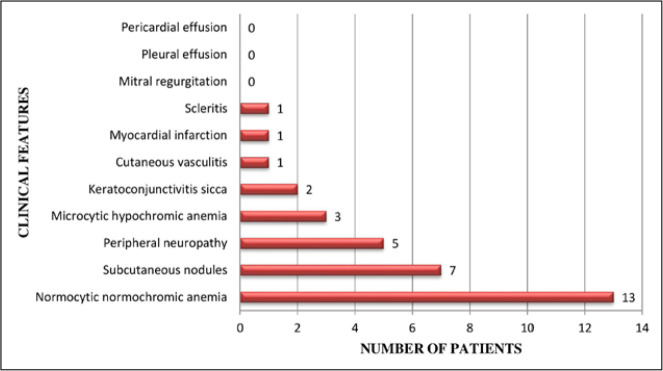
Extra-articular manifestation of double positive patients.

**Figure 3.**
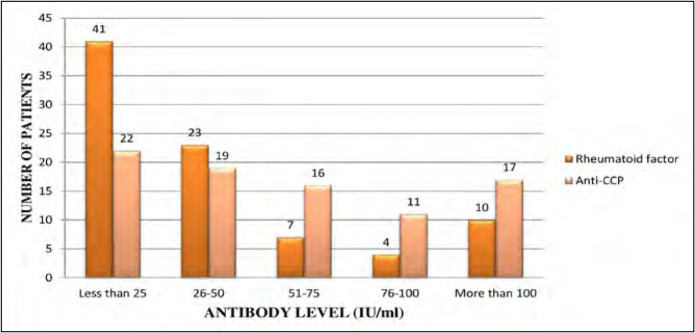
Titre values of RF and anti-CCP of RA patients.

Significant association was found between DAS 28 and anti-CCP (p=0.0026). Mean DAS 28 score in anti-CCP positive group was 5.15±1 and in anti-CCP negative group was 4.03±0.53 (p=0.0001) which was statistically significant. However, no such significant association was found between DAS 28 and RF, but the difference between mean DAS 28 in RF positive group (5.19±1) and RF negative group (4.97±1.03) was statistically significant (p=0.0001). Erosive disease (as seen on X-ray) was present in 48 patients (56%) as shown in **Table 1**. It was seen that more patients without erosions had disease activity <5.1 compared to the other group >5.1 and it was statistically significant (p=0.0021). It was also found that mean ESR and CRP was more in anti-CCP positive patients than anti-CCP negative patients (55 and 11 vs. 42 and 4). But both of which were statistically not significant (p=0.1526, p=0.1787). The various extra-articular manifestations along with their sex distribution are shown in **Figure 1**. On comparing the extra-articular manifestation (EAM) with anti-CCP positivity, we found that 39 patients with EAM were negative for anti-CCP, while 32 patients with EAM were positive for Anti-CCP but there was no statistically significant association between EAM and anti-CCP (p = 0.3762). From **Table 1** it is seen that patients with moderate to high disease activity had maximum EAM, while only 2 patients had EAM in low disease activity group. Despite this there was no statistically significant association between EAM and DAS 28 (p=0.377). Another finding that came out in our study was regarding the double positive patients. Out of 85 cases of RA we had 51 patients who were positive for both RF and anti-CCP. **Figure 2** depicts the extra-articular manifestation of those patients who were double positive (a few did not have any extra-articular manifestations). **Figure 3** depicts the titre values of RF and anti-CCP of RA patients.

Mean age of the total 105 AFDR was 28.54 ± 9.27 years with 1.02:1 male:female ratio. Anti-CCP sero-positivity was detected in 22 of 105 (21%) AFDR and in 13 of 105 (12%) control group respectively but the difference was not statistically significant (p = 0.137).

## DISCUSSION

RA is a multifactorial disease that has got a polygenic inheritance.^8^ Serum markers like anti-CCP and RF have been often used to favour the diagnosis of RA.^9^ Though the disease usually begins after 65 years,^10^ majority of our patients were between 40–50 yrs. This was comparable to the study done by Shankar S et al. in which the mean age was 41.6 ± 11.7 years.^11^ As RA is an autoimmune disease, the sex ratio in our study was more in favour of female supported by the findings of Fatima N et al. and Gabriel SE et al.^12,13^ The duration of RA of our patient pool was similar to study done by Aletaha and Ward, where it ranged from 2.5 months to 12.2 years.^14^ Symmetrical joint involvement was present in majority of our patients, and also, the joint involvement score was more in Anti-CCP positive patients which was supported by studies done by Cader et al. and Kim et al.^15,16^ The reason is that, joints under normal states do not contain citrullinated proteins, but different citrullinated proteins are present during various types of inflammation which occurs in RA.^17^ Thus, the antibody formed against them, directly affect the joint and their serum levels are used as marker of RA. Comparing the mean disease activity in our study with those of Serdaroglu M et al. (3.9 ± 1.3), Chen J et al. (4.4 ± 1.4), and Biomdo I et al. (3.68 ± 1.5), the present study had higher disease activity.^6,18,19^ On comparing overall disease activity using DAS28, with Anti-CCP and another comparison between Anti-CCP positive and negative group, our findings were statistically significant similar to the study done by Mansoor K et al.^20^ As similar results were not obtained for RF, it justifies the use of Anti-CCP over RF in supporting the clinical suspicion of RA and this was also supported by the study done by Yadollah S et al.^21^ Erosive disease in our study was less than what most studies had reported.^22,23^ Various reasons for erosion have been implicated. Increased production of RANKL and other cytokines, dysregulation of innate immune mechanisms and alterations of microRNA expression stimulate differentiation and function of osteoclasts, leading to bone erosions. Besides, increased levels of cytokines and overproduction of antagonists to the canonical Wnt signalling pathway with impaired production of bone morphogenetic proteins result in impaired osteoblast differentiation and function, deterring the capacity of bone erosions to repair^24^. So, the higher the disease activity, the higher the erosion is. Therefore, such group of patients would require frequent follow up and aggressive therapy to prevent irreversible joint damage. Though acute phase reactants like ESR and C-RP are more plentiful in Anti-CCP positive patients, there is no statistical significance difference from the Anti-CCP negative group; this finding was similar to the finding of Greiner et al.^25^ This is the reason due to which while calculating the DAS28 score, various parameters are taken into consideration, not just ESR, despite it being a strong predictor of inflammation. Study by Nicole CR et al supported our finding of no significant association of EAM and Anti-CCP positivity.^26^ Thus, it is recommended that clinicians should not rely on Anti-CCP for predicting the occurrence of EAM in RA patients. In contrary to the results of Lopez LG et al., there was no significant association between EAM and disease activity.^27^ However, a study by Desai et al. at Brigham and Women’s hospital had similar findings. Thus, predicting the severity of RA in a patient who comes with EAM is questionable and demands further clarifications. Though statistically not significant, AFDR had more prevalence of Anti-CCP than healthy controls. In the study by Seong KK et al., seropositivity for RF and Anti-CCP antibody was detected in 14.4% and 5.0% of unaffected FDRs respectively. Anti-CCP antibody seropositivity was more prevalent in FDRs in multi-case families (17.8%) when compared to those families with just single case (1.3%, p < 0.0001)^28^. Hani S et al. in their study showed the frequency of Anti-CCP antibodies was 82% in RA probands, 17% in FDR, 11% in more distant relatives, and 3% in controls.^29^ In the study by Goeldner I et al., the frequency of Anti-CCP was significantly increased in relatives of RA patients (5.5%; 11/200) when compared with healthy individuals (1%; 1/100; p=0.050).^30^ So, taking these findings into consideration, measuring serum Anti-CCP in FDR of RA patients may help in early detection of the disease, as these suspected cases will be closely followed up. Thus, early initiation of treatment shall help in achieving remission with minimum number of drugs at the lowest possible dose. The limitation we faced in our study was the small sample size, due to which despite getting a difference in the positivity of anti-CCP between AFDR and healthy controls the value did not come out to be significant. Thus, further studies with bigger sample size are warranted in this area.

## CONCLUSION

Anti-CCP antibodies should be tested at the earliest for RA because of their high specificity in RA. These antibodies are associated with more active, erosive, and severe form of RA. Its presence was not statistically associated with EAM. Thus, the group of the patients who are positive for Anti-CCP, need to be treated early and more aggressively in order to prevent irreversible joint damage. The higher sero-prevalence of Anti-CCP in AFDR in our study suggests that they may have a high risk of development of RA in the near future. Therefore, Anti-CCP positive AFDR require a long-term follow up for early detection of development of RA.

This project was a completely self-funded; no grant was availed for this research work.
